# The COMPASS-MS Study on Implementing Digital Assessment and Treatment for Psychological Distress in Patients With Multiple Sclerosis: Protocol for a Real-World Longitudinal Study

**DOI:** 10.2196/83416

**Published:** 2026-07-28

**Authors:** Emma Jenkinson, Rona Moss-Morris, Natasha Seaton, Eli Silber, David Okai, Abigail Wroe, Simon Brodie, Sam Norton, Joanna L Hudson

**Affiliations:** 1 Department of Psychology, Institute of Psychiatry, Psychology & Neuroscience, King's College London, Guy's Hospital, London, SE1 9RT, United Kingdom, +44 20 7188 0189; 2 Neurology Department, King's College Hospital, London, United Kingdom; 3 Lead Patient and Public Involvement Representative, London, United Kingdom; 4 Department of Inflammation Biology, Faculty of Life Sciences & Medicine, King's College London, London, United Kingdom

**Keywords:** digital health, mental health, depression, anxiety, psychological adjustment, psychological distress, multiple sclerosis, cognitive behavioral therapy, CBT

## Abstract

**Background:**

Comorbid anxiety and depression in patients with multiple sclerosis (MS) are common, conferring a greater risk of poorer outcomes and increased health care costs. Few MS services include scalable treatment pathways for psychological distress.

**Objective:**

This study aims to conduct a real-world longitudinal study evaluating the implementation and potential effectiveness of an integrated pathway involving digital screening for psychological distress and COMPASS-MS, a therapist-guided digital cognitive behavioral therapy tailored to the challenges of living with MS.

**Methods:**

This is a mixed methods, observational, real-world, longitudinal study being conducted in the United Kingdom. Routine mental health screening in the MS clinic will identify patients experiencing distress (using predefined clinical cutoffs), who will be assessed for eligibility for psychological treatment, including the COMPASS-MS program. Participants will receive COMPASS-MS online over approximately 12 weeks (including up to six 30-min therapist sessions). The implementation, reach, and adoption of the treatment pathway within a specialist MS service will be assessed using aggregate data on the uptake of mental health screening, eligibility, and consent rates for COMPASS-MS, as well as the number of COMPASS-MS sessions completed. Interviews with patients and health care professionals will primarily assess the scalability and potential or actual barriers and facilitators of the new pathway. Potential effectiveness will be assessed using participant questionnaires at preintervention and 12 weeks postintervention. The primary effectiveness outcome will be pre-post changes in distress (Patient Health Questionnaire Anxiety and Depression Scale scores). Quantitative data will be summarized using descriptive statistics and mixed effects models. Qualitative data will be analyzed using reflexive thematic and framework analysis.

**Results:**

Recruitment of both patients and health care professionals began in June 2024. For patients, recruitment was completed as of September 2025, including 82 participants. For health care professionals and stakeholders, as of September 2025, 7 had consented and completed interviews, and 13 more were expected to be recruited by August 2026. Data analysis has not yet started; however, quantitative results are expected by September 2026.

**Conclusions:**

The study findings will inform treatment pathways that can be incorporated into MS clinics and highlight adaptations or implementation protocols required to increase future scalability and effectiveness.

## Introduction

Multiple sclerosis (MS) is a chronic, incurable neurological condition characterized by the demyelination of the central nervous system. The majority of people living with MS are diagnosed with relapsing-remitting MS (~85%), which involves intermittent relapses or “flares” of new or existing symptoms, followed by periods of recovery or “remission” [1]. A smaller proportion, approximately 15%, have primary progressive MS [1]. Many with relapsing-remitting MS eventually transition to secondary progressive disease, after a median of 20 (range: 1‐51) years, in untreated cases [2]. People living with MS commonly experience diverse and unpredictable symptoms, including fatigue, mobility issues, and cognitive impairments. Unsurprisingly, psychological distress, including symptoms of depression and/or anxiety, is among the most common comorbidities in people living with MS [3]. Prevalence is estimated at 31% for depression and 22% for anxiety [4].

Psychological distress is associated with poorer clinical outcomes, including increased disease severity and disability [5,6], poorer quality of life [7], and reduced adherence to disease-modifying therapies [8]. It is also linked to increased health care usage and costs for health services [9]. Despite these consequences, MS treatment is largely focused on the biological aspects of the disease, and psychological comorbidities remain a substantial unmet need [10,11]. Evidence suggests that distress is often undetected or undertreated, with two-thirds of people living with MS who meet the criteria for depression receiving no medication or psychological support [12-15]. This gap persists despite the availability of reliable and brief screening tools, alongside expert consensus panels and people living with MS advocating for routine distress screening and support in MS clinics [16-18].

While both pharmacological treatment and psychotherapy show promise in addressing distress among people living with MS [19], evidence suggests that people living with MS prefer psychotherapy, demonstrating their inclination toward proactive support and skill-building to manage well-being [10]. Cognitive behavioral therapy (CBT) is recommended by the National Institute for Health and Clinical Excellence for treating depression and anxiety in the context of long-term conditions (LTCs), including MS [20]. CBT treatment protocols that are tailored to the challenges of living with MS appear to demonstrate larger treatment effects than traditional CBT [21], which reflects findings in other LTC populations, showing that LTC-tailored CBT increases treatment effect sizes for psychological distress alongside patient acceptability [22,23]. However, providing tailored CBT presents challenges for MS clinics due to cost, time, and workforce constraints.

Digitally delivered CBT presents a scalable solution. Digital CBT achieves similar effect sizes as face-to-face treatment while reducing costs and therapist time, as well as improving accessibility [24-27]. Researchers at King’s College London developed COMPASS, a therapist-guided, web-based CBT program designed to treat depression and/or anxiety in patients living with LTCs. COMPASS was based on the transdiagnostic model of adjustment to LTCs and targets mechanisms known to maintain depression or anxiety and prevent optimal psychological adjustment in people with LTCs [28].

The original transdiagnostic version of COMPASS was tested in a randomized controlled trial (RCT) compared with standard charity support [29]. This RCT, involving 4 distinct LTC populations (MS, inflammatory bowel disease, psoriasis, and kidney disease), demonstrated medium to large treatment effects on self-reported psychological distress, depression, anxiety, and illness-related distress across all LTCs at 12 weeks’ postrandomization [30]. However, exploratory moderation analysis suggested smaller effects for anxiety and illness-related distress in the MS subgroup compared with LTCs, indicating scope to further tailor the intervention for people living with MS and potential to boost the efficacy of COMPASS. Qualitative findings highlighted that people living with MS generally approved of COMPASS; however, they also desired more MS-specific content [31]. To address this, COMPASS was adapted for the population with MS (COMPASS-MS) using the person-based approach [32] and extensive patient and public involvement (PPI) [33]. A separate manuscript will detail the adaptation process [31].

Digital health tools are viable in MS care. For instance, a smartphone app for monitoring motor skills, mobility, and cognition was shown to be cost-effective through its impact on slowing disability progression, improving quality of life, and reducing medical costs [34]. To our knowledge, the cost-effectiveness of digital CBT tailored to patients with MS has not been assessed.

Moreover, while COMPASS shows promise, evidence of intervention effectiveness rarely translates to successful implementation; only 50% of psychological interventions from clinical research are embedded into routine usage [35], typically 17 years after development [36]. An existing service improvement project has investigated the implementation of a transdiagnostic version of COMPASS into a primary care, UK National Health Service (NHS) Talking Therapy setting [37]. However, the unique context and specific challenges for implementing COMPASS posed by a secondary care service for MS have not been explored. There is a need to evaluate COMPASS-MS not only for clinical effectiveness but also for implementation success, including barriers and facilitators to reach, adoption, acceptability, and/or scalability [38].

Qualitative research with people living with MS and health care professionals [39] suggests that effective mental health care for individuals with MS requires both internal service improvements (eg, better pathways for identifying and treating psychological distress) and external changes (eg, increased local funding). Developing and testing integrated pathways that include routine mental health screening and scalable, low-cost psychological support is an important step toward addressing these long-standing service gaps.

The overall aim of this mixed methods study is to evaluate the implementation and potential effectiveness of an integrated care pathway for treating psychological distress within 1 NHS neurology service for people living with MS. The integrated pathway includes routine mental health screening and COMPASS-MS, a digital therapist-supported CBT program adapted for MS. A nested qualitative study will additionally explore the barriers and facilitators to scaling the integrated pathway within and beyond the study site. In-depth interviews with stakeholders from the study site and approximately 6 to 8 additional neurology services will be conducted.

The study objectives were defined using 4 components of the RE-AIM (Reach, Effectiveness, Adoption, and Implementation) framework [40]. The specific objectives are as follows:

- Reach: to assess the sociodemographic and clinical reach of the new integrated care pathway in the MS clinic.
- Effectiveness: establish the potential effectiveness of the treatment pathway for people living with MS and the service.
- Adoption: to qualitatively explore the barriers and facilitators to adopting the integrated pathway from the perspective of patients and health care professionals at the study site, as well as stakeholders in other NHS neurology services.
- Implementation: to explore the potential implementation of COMPASS-MS in routine practice, including engagement, adherence, and therapist fidelity.

## Methods

### Study Design

This real-world longitudinal, single-arm study includes 2 nested qualitative studies. Quantitative data will be collected at pretreatment and posttreatment (12 wk). This protocol is aligned with the SPIRIT (Standard Protocol Items: Recommendations for Interventional Trials) guidelines (Checklist 1), which has been adapted for single-arm, nonrandomized studies.

### Study Setting

This study will be conducted within a London-based outpatient neurology MS clinic. The MS clinic is a specialist service and provides care for approximately 1000 adults with MS who are aged 18 years or older. The clinic employs 5 consultants, 5 nurses, and 4 community MS nurses. Currently, psychological provision within the service is limited; however, the medical team liaises with a neuropsychiatrist who advises the team on addressing mental health needs.

### Study Participants

Patients with a diagnosis of MS, aged 18 years or older, who are experiencing psychological distress will be eligible for inclusion. Mental health screening and treatment will be available to patients who have an appointment at the MS clinic during the study’s implementation.

### Routine Care: Screening and Treatment of Psychological Distress

#### Psychological Screening

In the current standard of care, psychological distress is identified through informal conversations between patients and clinicians during consultations. This process may result in a direct referral.

The novel pathway introduced as part of this study will include routine screening for depression and anxiety, delivered via MyChart (Epic) [41]. MyChart is a mobile app that is integrated into the hospital’s electronic health records. MyChart digitally collects patient-reported outcome measures (including depression and anxiety) within outpatient services. Therefore, all patients who have downloaded the MyChart app will be prompted to complete digital mental health screening prior to their appointment. Patients will not be able to complete the screening if they do not have a registered MyChart account accompanying their patient record. MyChart screening results appear in patients’ electronic health records so that clinicians can review them, discuss them, and refer as appropriate.

For the implementation of this pathway, the clinical team will use trainee psychologists and therapists to complete psychological assessments, supervised by a clinical and health psychologist (AW), and a consultant neuropsychiatrist and CBT therapist (DO). All patients who demonstrate mild symptoms of distress (scores ≥5 on the Patient Health Questionnaire-8 [PHQ-8] [42] for depression or the Generalized Anxiety Disorder-7 [GAD-7] scale [43] for anxiety) through MyChart, as well as all patients directly referred by clinicians, will be assessed over the telephone.

**Figure 1.**
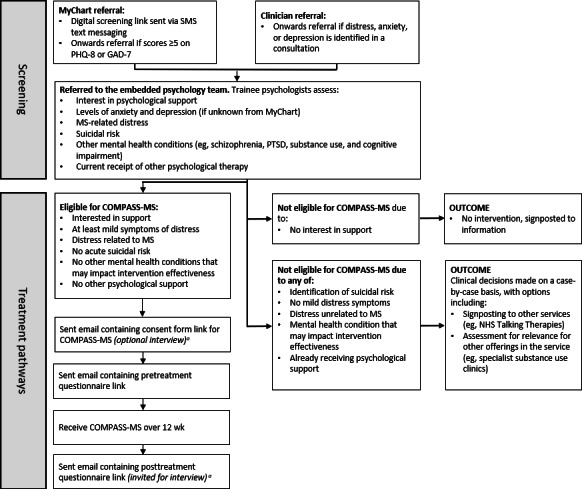
Referral, screening, and treatment pathway overview. ^a^The consent form will allow participants to opt in to a posttreatment interview. Participants were purposively sampled for the posttreatment interviews to capture the range of sociodemographic diversity and engagement with COMPASS-MS. GAD-7: Generalized Anxiety Disorder-7; MS: multiple sclerosis; NHS: National Health Service; PHQ-8: Patient Health Questionnaire-8; PTSD: posttraumatic stress disorder.

Using a trainee psychologist model within the service will enable the new pathway to be delivered with minimal additional resources and cost. The assessment process aims to identify 6 key aspects (Figure 1). Briefly, participants are eligible for COMPASS-MS if they are interested in receiving therapy, exceed distress cutoffs (PHQ-8 or GAD-7 ≥5), report MS-related distress, and show no active suicidal ideation. Patients not meeting these specific criteria and seeking support are signposted to internal neurology service offerings or external services, for example, talking therapy services.

#### COMPASS-MS Treatment

##### COMPASS-MS Overview

The theoretical underpinnings and full description of the original COMPASS intervention are reported elsewhere [28,29]. In brief, COMPASS is a web-based, therapist-supported CBT intervention delivered over 12 weeks. Patients can complete 11 online modules and receive up to six 30-minute therapist support sessions delivered over telephone, videoconferencing, or in-site messaging. The online modules consist of psychoeducation, patient stories, goal setting, and interactive tasks that use evidence-based CBT techniques. The modules explore a range of topics, such as managing the uncertainty of living with an LTC, managing unhelpful thoughts and symptoms, and using social and professional support. In COMPASS-MS, certain aspects, such as the patient stories, have been adapted to include lived examples of common challenges experienced by patients with MS [31]. However, the basic components, delivery, and CBT techniques remain the same as the original COMPASS intervention. Therapists will be clinical or health psychology trainees and will have at least basic CBT training, alongside COMPASS-specific training, an intervention manual, therapeutic guidance for the digital platform, and standard operating procedures for suicidal risk (see Multimedia Appendix 1 for service procedures). Specifically, if a participant indicates active suicidal ideation or intent, a risk protocol will be triggered. This involves conducting a detailed risk assessment, contacting the general practitioner, and establishing a personalized safety plan, which may include signposting, contacting next of kin, and/or referral to emergency or crisis services. If risk is identified during a therapist session, the therapist is responsible for initiating the risk pathway. If risk is identified during an assessment on COMPASS, the COMPASS intervention automatically notifies the risk lead, triggering an immediate response from the research team. Therapists will receive weekly supervision with a clinical and health psychologist (AW) and/or a neuropsychiatrist (DO).

##### COMPASS-MS Intervention Inclusion Criteria

Patients will be eligible for COMPASS-MS if they are 18 years or older, have a diagnosis of MS, possess sufficient English language skills to interact with digital CBT, have access to a computer or device to complete the intervention, present with at least mild symptoms of distress, and demonstrate evidence of illness-related distress.

Patients will not be eligible if they have an active suicidal risk (recent suicidal intent and/or plans); are currently receiving psychological treatment; have a mental health presentation that is not related to MS; or have a health issue identified by a clinician that may be a barrier to using COMPASS-MS, such as substance dependency, a severe mental health condition (such as posttraumatic stress disorder), or severe cognitive impairment.

### Study Procedures

#### Procedure: Patient Participants

The route of routine screening (MyChart vs clinician referral) and whether a patient completes a psychological assessment will be pseudoanonymized, recorded, and shared with the research team. For patients who complete a psychological assessment, demographic information (age, gender, ethnicity, and index of multiple deprivation) and clinical information (depression, anxiety, cognitive impairment, and disability) will be collected to determine the appropriate treatment pathway (more details are given in the “Implementation Measures” and “Effectiveness Measures” sections).

Following patient screening and assessment, eligible and interested patients who have consented to share their contact details will be contacted by the research team. Patients will be emailed the information sheet and a link to the informed consent form. Upon receipt of consent, a link to the baseline questionnaire (pretreatment) will be sent. Following its completion, patients will be enrolled in COMPASS-MS. Patients will complete a follow-up questionnaire 12 weeks after baseline (posttreatment). All recruitment procedures will be conducted remotely via telephone or the Qualtrics online platform [44]. Pre- and posttreatment questionnaires will also be administered through Qualtrics. These questionnaires will assess the potential effectiveness of COMPASS-MS. Additionally, eligible patients will have the option to consent to participate in a qualitative interview study. A subgroup, purposively sampled to capture sociodemographic diversity and different levels of engagement with COMPASS-MS, will be invited to take part in an interview about their experience with the integrated pathway.

#### Nested Study Procedure: Interviews With Stakeholders and Health Care Professionals

##### Overview

Stakeholders within the NHS trust at the study site will be approached to take part in the qualitative interview study via email. The email will include a study information sheet and a link to a consent form to be filled online via Qualtrics. Stakeholders outside of the study site will be identified based on the geographic location of their services. This approach will enable a representative understanding of the challenges experienced across the country. The site principal investigator (ES) and senior author (RM-M) will identify appropriate services. Following this, the research team will approach the services with information about the study and will direct any potential stakeholder participants who express interest back to the central research team.

##### Nested Qualitative Interview Study: Inclusion Criteria

Eligible stakeholders will be those who engaged with the new care pathway at the study site, specifically those who have been involved in routine mental health screening, direct referrals to psychology, assessing patients as part of the new pathway, and providing COMPASS-MS therapist support. Furthermore, other stakeholders who work with people living with MS in the study site (eg, physiotherapists, occupational therapists, or commissioning bodies) will be eligible for inclusion.

Additionally, individuals who work with people living with MS in NHS trusts outside of the study site will also be eligible, including doctors, nurses, psychologists, occupational therapists, physiotherapists, and members of commissioning bodies. This will broaden the scope to include barriers and facilitators to implementation outside of the study site.

### Outcomes

Coprimary objectives are to explore the real-world implementation and effectiveness of a new integrated care pathway (mental health screening and COMPASS-MS treatment) in a specialist MS service, which treats approximately 1000 patients with MS. Our outcomes were defined using 4 components of the RE-AIM framework [40]. The specific outcomes are as follows:

#### Reach

Reach will describe the population who participate in the intervention. This will include the number, percentage, and characteristics of people living with MS who:

- Complete routine screening and psychological assessment.
- Report clinical levels of psychological distress at assessment. Associations between distress and demographic or clinical characteristics will also be assessed.
- Meet the inclusion criteria for COMPASS-MS and consent to the COMPASS-MS study? Reach will also be assessed by comparing the demographic characteristics of patients who were referred to the study and those who consented to participate in COMPASS-MS.

#### Potential effectiveness

Potential positive impacts of the intervention on important outcomes will be assessed, at the level of the patient. These outcomes include:

- What are the changes pre-post COMPASS-MS treatment for self-reported clinical outcomes (the primary effectiveness outcome is the change in psychological distress from baseline to 12-weeks, measured with an anxiety and depression composite score)? Additionally, secondary effectiveness outcomes will include depression, anxiety, illness-related distress, functioning, and quality of life).
- What are the changes pre-post COMPASS-MS in healthcare utilisation to explore potential cost-effectiveness?
- What are the potential mechanisms of change pre-post COMPASS-IBD (cognitive and behavioural responses to symptoms, acceptance of illness, intolerance of uncertainty, expression of emotion, health behaviours)?

The service-level impact of the intervention will also be assessed. These outcomes include:

- How many people living with MS are subsequently identified as requiring more intensive treatment input after COMPASS-MS?
- What are the demographic and clinical characteristics of people living with MS who require more intensive treatment input following COMPASS-MS?

#### Adoption

Adoption will be explored *qualitatively* through themes on barriers and facilitators from patient and stakeholder interviews. Specifically:

- What are the barriers and facilitators to *screening* and *treating distress using COMPASS-MS* from the *perspective of patients with MS and stakeholders* exposed to the care pathway?
- What are the barriers and facilitators to *scaling the pathway* to sites outside the study site?

#### Implementation

Implementation will explore how closely the intervention is received as intended. It will be assessed with the following questions:

- What are the rates of adherent users, nonengagers, and dropouts? For adherence, we will report percentages of those adhering to the online component, the therapist component, and meeting the composite adherence definition (see the “Implementation” subsection in the “Implementation Measures” section).
- What is the therapist’s fidelity to the COMPASS-MS treatment?

### Implementation Measures

#### Reach

Data collected during screening and triage to determine appropriate treatment pathways will include the number, percentage, and characteristics of patients who (1) agree to and complete routine screening for psychological distress via MyChart; (2) receive a direct referral from a clinician for psychological distress after the identification of distress during a consultation; (3) complete psychological assessment; (4) are interested in receiving psychological support; (5) report clinical levels of psychological distress, depression, or anxiety at the time of assessment, and their sociodemographic predictors of these outcomes also evaluated; (6) report their distress as related to their experience with MS; (7) report the presence of active suicidal risk; (8) express interest in participating in the COMPASS-MS study; (9) meet the inclusion criteria for COMPASS-MS and provide consent to participate in the COMPASS-MS study, evaluating differences in demographic characteristics compared with those referred; and (10) receive COMPASS-MS but are subsequently identified during treatment as requiring more intensive treatment.

The characteristics that will be evaluated are as follows: (1) demographic information (age, gender, ethnicity, and index of multiple deprivation); (2) depression and anxiety measured using the PHQ-8 [42] and GAD-7 [43], respectively; (3) mild cognitive impairment assessed using the Montreal Cognitive Assessment telephone version [45,46]; and (4) Expanded Disability Status Scale score [47], which is clinician-reported based on an examination by a neurologist, with ranges from 0 to 10 in 0.5-unit increments, with higher scores reflecting greater disability.

For those who consent and complete the pretreatment questionnaire, data will be gathered on sociodemographic factors (age, gender, ethnicity, and index of multiple deprivation) and clinical factors (MS type, year of diagnosis, comorbidities, commonly occurring MS symptoms, relapse history in the last 12 wk, and medication to treat relapses).

#### Adoption

Patient interviews will be conducted remotely and will follow a semistructured interview guide. The interviews will focus on the experiences of people living with MS regarding MyChart mental health screening and clinician referral for support, study participation, and using COMPASS-MS.

Stakeholder interviews will assess perceptions of mental health screening and the treatment of psychological distress in people living with MS, as well as any barriers and facilitators to these processes. Additionally, for those exposed to MyChart screening and COMPASS-MS treatment, the interviews will explore the perception of implementing the integrated care pathway. The interview guide will be informed by the Normalization Process Theory [48] and the NASSS-CAT (Non-Adoption, Abandonment, and Challenges to Scale-up, Spread, and Sustainability) tool [49]. Stakeholder interviews will be conducted in-person or remotely.

#### Implementation

To explore the potential implementation of COMPASS-MS, we will record the following. First, the number of patients requiring digital support to use COMPASS-MS. Second, the number of online sessions completed and the time spent engaging with COMPASS-MS sessions (in minutes). This usage data will be collected and automatically recorded on the COMPASS-MS web platform. Third, the number of sessions attended, the length (in minutes), and the mode of session delivery for therapist sessions will be recorded manually by the therapist. Fourth, health service outcome on completion of COMPASS-MS, such as dropout. Finally, therapist fidelity to the COMPASS-MS treatment will be assessed. Telephone and videoconferencing therapist sessions will be audiorecorded and assessed by the clinical and health psychologist supervisor (AW). A bespoke fidelity scale will be used to check that therapists are using the relevant CBT skills and adhering to the protocol. This fidelity scale has been used previously [29]. Regular supervision (AW and DO) will provide support for therapist fidelity to the treatment.

We will report rates of engagement and adherence, as well as the demographic and clinical characteristics of the following groups: (1) nonengagers (those who are enrolled, but never use COMPASS-MS), (2) less-adherent users (those who use COMPASS-MS but participate in <5 online sessions and receive <3 support sessions), (3) those adhering to the online component (those who attend ≥5 online sessions), (4) those adhering to the therapist component (those who attend ≥3 appointments), and (5) fully adherent users (those who complete ≥5 online sessions and attend ≥3 appointments).

### Effectiveness Measures

#### Primary Patient Effectiveness Outcome

Psychological distress will be measured pretreatment and posttreatment using the PHQ Anxiety and Depression Scale (PHQ-ADS) [50], which combines the PHQ-9 (9-item version) [51] and GAD-7 [43] into a 16-item measure of distress. A decrease of 4 or more points on the PHQ-ADS is considered a minimum clinically important difference [52], and total score cutoffs of 10, 20, and 30 indicate mild, moderate, and severe levels of distress, respectively [52].

#### Secondary Patient Effectiveness Outcome

Five secondary effectiveness outcomes will be collected as follows. First, *anxiety* will be measured using the GAD-7 [43], a 7-item self-report anxiety questionnaire. The questionnaire has a scale range of 0 to 21; higher scores indicate increased anxiety symptoms. Second, *depression* will be measured using the 9-item self-report PHQ [51]. This questionnaire has a scale range of 0 to 27; higher scores indicate increased depressive symptoms. Third, *illness-related distress* will be measured using the Illness-Related Distress scale [53]. The Illness-Related Distress scale is composed of two 7-item subscales (interpersonal and intrapersonal distress) answered on a 4-point scale. Each subscale ranges from 0 to 21, with higher scores indicating greater distress. Fourth, *social functioning* will be measured using the Work and Social Adjustment Scale, a 5-item measure of functional impairment [54]. The Work and Social Adjustment Scale has a scale range of 0 to 40, with higher scores indicating greater functional impairment. Finally, *health-related quality of life* will be measured using the EuroQol 5-Dimension Scale (EQ-5D-3L) [55]. The EQ-5D-3L includes 5 dimensions (range of scores, 1-3) that assess mobility, self-care, usual activities, pain or discomfort, and anxiety and depression. High item scores indicate poorer quality of life. The EQ-5D-3L also includes a visual analog rating scale (range: 0‐100), whereby higher scores reflect better global health.

Moreover, to assess potential cost-effectiveness, patients will be asked to self-report their health care usage. *Health service usage* will be explored through the 4 service use items from the Client Service Receipt Inventory [56]. These items ask participants to self-report whether they have visited their general practitioner, accessed psychologist or therapist support (outside of the study), visited a secondary care service, and accessed emergency care in the last 3 months.

#### Process Variables

Additionally, secondary outcomes will include process variables as potential mechanisms of change to understand the underlying intervention effects. These will include the following measures:

- *Cognitive and behavioral responses to symptoms* will be measured using the Cognitive and Behavioural Responses Questionnaire—Short Form (CBRQ-SF). This study will use an adapted version of the CBRQ-SF [57], including 5 subscales from the CBRQ-SF: symptom focusing (3 items), damage beliefs (3 items), embarrassment avoidance (3 items), all-or-nothing behavior (3 items), and fear avoidance (3 items) subscales. Additionally, we will include the catastrophizing subscale (4 items) from the original CBRQ [58]. Items are rated on a 5-point Likert scale ranging from “strongly disagree” to “strongly agree.” Each of the subscales in the shortened and original CBRQ have been shown to be reliable and valid [57,58].
- *Acceptance of illness* will be measured using the Acceptance of Chronic Health Conditions Scale, which has demonstrated reliability and validity in chronic conditions [59]. This 10-item scale scores items on a 5-point Likert-scale from 1 (“strongly agree” to 5 (“strongly disagree”). Items were adapted to include “MS” in the wording. Items pertaining to positive attitudes toward the condition are reverse scored. Higher scores therefore indicate higher levels of acceptance of the condition.
- *Intolerance of uncertainty* will be measured using the Intolerance of Uncertainty Scale-12 item [60]. The 12-item scale is rated on a 5-point Likert scale ranging from 1 (“not at all characteristic of me”) to 5 (“entirely characteristic of me”). Items on the questionnaires are summed, and therefore scale scores can range from 12 to 60.
- *Expression of emotion* will be measured using the Beliefs about Emotions Scale [61]. This 12-item scale assesses beliefs about the unacceptability of expressing and experiencing negative emotions. Each item is responded to on a 7-point Likert scale ranging from 0 (“totally disagree”) to 6 (“totally agree”). The scale is scored by summing the items, and thus the total scores can range from 0 to 72. High scores on the scale represent a greater belief that it is unacceptable to express emotions.
- *Health-related behaviors*, such as smoking status and alcohol use (average weekly consumption), will also be recorded.

### Sample Size

The target patient sample size for the study, consisting of 75 participants for the COMPASS-MS intervention, was determined based on pragmatic considerations of the number of people living with MS attending the MS service and the prevalence of distress. The objective of the mental health screening is to assess all eligible people living with MS attending the MS clinic during the study period. However, we conservatively estimate a 50% consent rate for screening, resulting in 500 out of 1000 patients completing the screening. Based on findings from Boeschoten et al [4], we anticipate that 30% of these screened patients (n=150) will experience distress. If 50% of these distressed patients find the treatment acceptable, we will achieve our target sample size of 75.

As this is a real-world implementation study, a formal power calculation was not performed; however, a sample of 75 provides sufficient precision to estimate implementation metrics and detect a moderate within-group effect size (Cohen *d*=0.32‐0.35) on the primary outcome with 80% power at an α level of .05. We acknowledge that the study may be underpowered for moderator analyses or subgroup comparisons, particularly if attrition is moderate.

### Statistical Methods and Data Analysis

Most Reach and Implementation outcomes will be summarized descriptively. Categorical variables will be summarized with frequencies and percentages, while continuous variables will be reported as means and SDs or as medians with IQRs, depending on the distribution. Where relevant, rates (eg, eligibility, adherence) or mean differences (eg, change from baseline) will be presented alongside 95% CIs to quantify uncertainty in estimates. In the screened sample, differences in psychological distress, depression, and anxiety between key demographic characteristics (age, gender, and ethnicity) and any interaction effects will be examined using linear regressions. For between-group comparisons (eg, demographic and clinical differences between referred patients vs those who consent to COMPASS-MS and COMPASS-MS participants with baseline data only vs COMPASS-MS participants with complete data), chi-square test, Mann-Whitney *U* test, and two-tailed independent sample *t* tests will be conducted, depending on the distribution of the variable.

Potential effectiveness will be assessed using mixed effects linear regression, including a random intercept to account for repeated measurements within participants. Assessment time points will be modeled as dummy-coded covariates to estimate change from baseline, adjusting for demographic and relevant clinical variables. Interaction terms between time and covariates will be tested to explore whether the change from baseline differs across subgroups. Effect sizes will be reported as standardized mean differences, using the adjusted mean change from baseline from the mixed-effects models and the pooled SD (treating baseline as the control). Mixed-effects models will handle missing data using full-information maximum likelihood, under the missing-at-random assumption. Missing item-level data within self-report measures will be handled using proration when at least 50% of items are completed.

A per-protocol sensitivity analysis will be undertaken for potential effectiveness outcomes, restricted to participants who fully adhered to the treatment protocol (≥5 online sessions and attending ≥3 appointments). Moreover, moderator analyses are exploratory and will be conducted for the primary effectiveness outcome with the intention-to-treat sample to examine the heterogeneity of the treatment effect across MS subtype, age, gender, ethnicity, and education level.

To address the risk of type I error due to multiple testing of secondary outcomes and mechanisms, we will correct for multiple statistical comparisons by using a Holm-Bonferroni correction method. The interpretation of findings will prioritize the primary effectiveness (PHQ-ADS) and core RE-AIM implementation outcomes to ensure a focused evaluation of the intervention. Long-term follow-up beyond 12 weeks is not part of this study design; however, service-level reach data will be tracked over an 18-month implementation period.

Qualitative data will be analyzed using an inductive thematic analysis for the interviews with stakeholders, incorporating an iterative constant comparison approach. Conversely, patient interviews will be analyzed using a framework technique to enable comparisons with other COMPASS studies (eg, Jones et al [62]). Coding will be completed line-by-line by members of the research team, with regular discussions to refine coding frameworks and record analytic reflections. Interviews with patients and health care professionals will be analyzed and reported separately. Data collection and analysis will occur concurrently to allow emerging insights to inform subsequent interviews. NVivo software (Lumivero) will be used to facilitate data management and analysis.

### PPI

The person-based approach [32] was central to the original development of COMPASS, as described in detail elsewhere [28,29]. PPI continues to play a central role in this project, with our lead PPI representative actively contributing to study design, intervention development, data analysis, interpretation of findings, and dissemination activities.

To support the MS-specific adaptation of COMPASS, we convened a PPI advisory group comprising 10 members: 8 people living with MS (including the lead representative) and 2 health care professionals. This group contributed to the co-design process through structured workshops and targeted consultations, ensuring that the intervention content and delivery were relevant to the lived experiences of people living with MS. Clinicians within the MS service were also regularly consulted to refine the integrated treatment pathway during the study’s planning and implementation phases. Further details of the PPI activities undertaken in this project, including the impact of patient and public contributions, will be reported in subsequent publications.

### Ethical Considerations

The study has been approved by the Health Research Authority and Health and Care Research Solihull (Research Ethics Committee reference 24/WM/0085). All participants will provide written, informed consent. COMPASS-MS has been developed in accordance with the King’s Digital Therapies Quality Management System, ensuring compliance with the Medical Devices Directive 93/42/EEC for class I medical devices. It adheres to all clinical safety and quality management protocols, including supervision by a digital clinical safety officer.

### Dissemination

The results from the study will be published in peer-reviewed journals and will be presented at relevant conferences. Additionally, the findings may be disseminated through social media, web posts, and live events. A newsletter summarizing the study findings will be disseminated to participants who indicate a wish to receive it.

## Results

Recruitment of both patients and health care professionals began in June 2024. For patients, recruitment was completed as of September 2025, including 82 participants. For health care professionals and stakeholders, as of September 2025, 7 had consented and completed interviews, and 13 more were expected to be recruited by August 2026. Data analysis has not yet started; however, quantitative results are expected by September 2026.

## Discussion

### Principal Findings

This protocol outlines the rationale, design, and methods for evaluating the implementation of a new integrated care pathway through which psychological distress is digitally screened, and then treated with a digital CBT program adapted for MS (COMPASS-MS) within an NHS neurology service. The study will gather data from both patients and health care professionals to examine the reach, adoption, and implementation of this integrated care pathway, as well as the potential effectiveness of COMPASS-MS in real-world practice. This will be the first study to assess the delivery of COMPASS-MS in routine clinical care, evaluating both patient-level changes in psychological distress and broader service-level outcomes.

Findings from this research will inform how such a pathway could be embedded into other NHS MS services. By increasing access to low-cost digital CBT, this model has the potential to expand psychological support provision without substantial additional resource demands. The results will generate practical recommendations for future roll-out, while recognizing that each service context will present its own challenges in relation to workforce capacity and access to psychological supervision.

Moreover, this study may have important implications regarding the introduction of routine mental health screening via software such as MyChart. While screening is intended to ensure that those with psychological needs are identified and supported, it may also uncover unmet demand, increasing the number of patients requiring intervention. However, incorporating lower-intensity, scalable options such as COMPASS-MS could help address this by providing timely support to patients experiencing moderate distress while reserving more resource-intensive face-to-face therapy for those with greater clinical need.

Insights from this single-site study will be valuable in planning a larger, multicenter implementation trial. For example, a centralized “hub” model, where specialist therapists provide support across multiple NHS sites, may facilitate equitable access and consistent delivery. It will also be important to explore how the pathway can be adapted to settings with varying levels of existing psychological provision, including those with limited capacity for digital screening or in-house supervision.

Finally, beyond the immediate MS context, the findings from this study have potential relevance for integrating mental health screening and digital psychological interventions into other LTC outpatient pathways. Disseminating the results to national and local stakeholders will be critical for identifying opportunities to scale and sustain this model of care across the NHS.

### Limitations

This study employs a single-arm, observational design within 1 specialist MS clinic conducted within a specific UK NHS context. The lack of a control group means that observed changes in distress cannot be definitively attributed to the intervention, as natural symptom fluctuation or regression to the mean may occur. Moreover, findings may have limited generalizability to different MS service models across the United Kingdom or to international health care systems with varying structures and resources. Furthermore, the 12-week follow-up period precludes assessment of the long-term sustainability of clinical benefits or implementation success. Future multicenter RCTs will be required to confirm these preliminary findings.

## Supplementary material

10.2196/83416 Multimedia Appendix 1 Supplementary material containing the risk standard operating procedures.

10.2196/83416 Checklist 1 SPIRIT checklist.

## Abbreviations

CBRQ: Cognitive and Behavioural Responses Questionnaire
CBT: cognitive behavioral therapy
EQ-5D-3L: European Quality of Life Scale
GAD-7: Generalized Anxiety Disorder-7
LTC: long-term condition
MS: multiple sclerosis
NASSS-CAT: Non-Adoption, Abandonment, and Challenges to Scale-up, Spread, and Sustainability
NHS: National Health Service
PHQ-8: Patient Health Questionnaire-8
PHQ-ADS: Patient Health Questionnaire Anxiety and Depression Scale
PPI: patient and public involvement
RCT: randomized controlled trial
RE-AIM: Reach, Effectiveness, Adoption, and Implementation
SPIRIT: Standard Protocol Items: Recommendations for Interventional Trials
