# Supplementary material for: The COMPASS-MS Study on Implementing Digital Assessment and Treatment for Psychological Distress in Patients With Multiple Sclerosis: Protocol for a Real-World Longitudinal Study
Source: JMIR Res Protoc. 2026 Jul 28;15:e83416. doi: 10.2196/83416 (PMC13411437; doi:10.2196/83416)
Supplement: Multimedia Appendix 1 [file resprot-v15-e83416-s001.docx]

Supplementary Material: A real-world longitudinal study to implement digital assessment and treatment for psychological distress in multiple sclerosis (MS): The COMPASS-MS study protocol

## Supplement 1: Risk Standard Operating Procedures

**PROTOCOL FOR ASSESSING AND REPORTING RISK IN RESEARCH**

**The COMPASS-MS study**

The following principles and procedures govern risk assessment and reporting.

**General procedures**

Whenever any significant risk is identified a risk assessment should be completed and (counter signed) by the principal investigator and/or nominated deputy as soon after the assessment as possible.

Any significant, but not imminent risk should be reported to the person’s GP and, if appropriate, other health care professionals, as soon as is reasonably possible.

Any imminent risk should lead to the immediate involvement of the appropriate emergency health services. The principal investigator must ensure that Research Associates, Assistants, and Students are fully informed and competent to follow the procedures.

When the principal investigator is away they should ensure appropriate cover is arranged for any risk issues that might arise in their absence.

**Exploring Risk**

There are seven questions to be used following any indication of risk from responses to interview questions or any other sources. We define risk as any reporting active plans of suicide/self harm.

Ask the **Exploring Risk in Research Interviews questions** below and then look at answers from the sheet to determine the level of risk, A, B, or C.

**Exploring Risk in Research Interviews/Item 9 on PHQ 9**

The below risk assessment script and related actions should be performed when:

- Active suicidal plans are disclosed during a patient interview

OR

- The patient indicates the below response on Item 9 on the PHQ9 and associated follow-up risk questions during their 0, 12wk questionnaire:
  - PHQ9 – Item 9 score of 1 or more

AND

- - Answers YES to extra risk question *“Are you currently making plans about how you would end your life?”*

**Assessment script**

**THOUGHTS**

*“I see that you’ve said / you mentioned that……...*

*These are thoughts / feelings that people suffering from depression often have, but it’s important to make sure you are receiving the right kind of support. So if it’s OK, I would now like to ask you some more questions that will explore these feelings in a little more depth.”*

**PLANS**

1. Have you made any actual plans to end your life? Yes / No

If **yes –** details

**ACTIONS**

1. Have you made any actual preparations to kill yourself? Yes / No

If **yes –** details

1. Have you ever attempted suicide in the past? Yes / No

If **yes –** details

**PREVENTION**

1. Is there anything stopping you killing or harming yourself

at the moment? Yes / No

If **yes –** details

1. Do you feel that there is any immediate danger that you

will harm or kill yourself? Yes / No

Details:

**Researcher Risk Protocol**

Look at answers from the sheet to determine the level of risk, A, B1, or C:

| **Actions by Researcher** | ***Tell Participant*** |
| --- | --- |
| All answers ‘no’ apart from Q4 ‘yes’:  **A** | *I can see that things have been very difficult for you, but it seems to me these thoughts about death are not ones you would act on – would this be how you see things? (if they say yes) I would advise you to make an appointment to see your GP to talk about these feelings and I will write to them also to inform them of our discussions. In the meantime, I will email you a list of helpful contacts for your area should anything change so that you can get some more immediate support.* Send letter to GP. |
| ‘Yes’ for any **one** of Qs 1-3; plus ‘yes’ for Q4 and ‘no’ for Q5  **B1** | *Things seem to be very hard for you right now and I think it would help if you were to speak to your GP about these feelings. I will be writing to your GP to tell them that we have spoken and that you have been having some troubling thoughts. I would also advise you to make an appointment to see your GP to talk about these feelings. In the meantime, I will email you a list of helpful contacts for your area should anything change so that you can get some more immediate support.*  Send letter to GP.  **Complete a Safety Plan with patient:**  Establish a personalised Safety plan with patient when risk of suicide is detected following the steps below:   1. **What can you do to take care of yourself?**   This could include things like 'write down how I feel', 'cuddle a pet' or 'do some exercise like walking or swimming'.   1. **How would you like to be supported?**   Try to prod for a list of names and numbers of friends, family or professional's patient can contact when they need support and details of how they would like to be supported, like 'ask me how I'm feeling' or 'come to appointments with me'. It's also a good idea to list the details of helplines or peer support groups, which might include online support like Mind's Side by Side.   1. **Who can be contacted in an emergency?**   It's best to agree on [what to do in an emergency](https://www.mind.org.uk/information-support/guides-to-support-and-services/crisis-services/accident-emergency-a-e/), with names and numbers for [crisis services](https://www.mind.org.uk/information-support/guides-to-support-and-services/crisis-services/accident-emergency-a-e/). This could include things like 'call 999 for an ambulance', 'contact my Community Mental Health Team (CMHT) on...' or 'call 116 123 for Samaritans'. |

| Scoring ‘no’ to Q4 or ‘yes’ to Q5  **C** Actively Suicidal | *I am very concerned about your safety at this moment. I am not qualified to deal with this on my own as I am not a therapist, but I would like you to talk to one right now. I am going to contact someone* to let them know how you are feeling and to arrange for you to receive immediate help. Send letter to GP, call 999 and explain situation, request a welfare check. |
| --- | --- |

| Action to take in the case of immediate risk:  Participant needs immediate help – **do not leave them alone**. If on a telephone call with the participant ask them to stay on the line while you arrange suitable care.  Follow the chain of supervisory clinical contact (only one contact needed from 1-2) and enact immediate risk procedure (point 3):   1. Lead researcher 2. Principal Investigator (KCL) 3. 999 4. Clinical and Health Psychologist (AW) 5. Psychiatrist (DO) 6. Site Principal Investigator (ES) |
| --- |
|  |
